# Thermal Analysis of 1,6-Hexanediol Diacrylate-Based Green Bodies Containing GYAGG:Ce Fabricated by DLP 3D Printing

**DOI:** 10.3390/polym18202554

**Published:** 2026-10-16

**Authors:** Lydia V. Ermakova, Ramil R. Khasbiullin, Rasim R. Saifutyarov, Elizaveta V. Ermakova, Petr S. Sokolov

**Affiliations:** 1 National Research Center “Kurchatov Institute”, 1 Akademika Kurchatova Square, 123098 Moscow, Russia; rosya01@gmail.com; 2 Frumkin Institute of Physical Chemistry and Electrochemistry, Russian Academy of Sciences, 31-4, Leninsky Prospect, 119071 Moscow, Russia; khasbiullin@techno-poisk.ru (R.R.K.); dr.evermakova@phyche.ac.ru (E.V.E.)

**Keywords:** acrylate monomer, additive manufacturing, photosensitive resins, thermal analysis

## Abstract

The thermal decomposition of DLP-printed green bodies containing a cross-linked 1,6-hexanediol diacrylate matrix filled with complex oxide powder of a cerium-activated garnet structure (GYAGG:Ce) was investigated. Non-isothermal thermogravimetric measurements were performed under an argon atmosphere at heating rates of 2, 4, 8 and 20 °C/min (four independent measurements for each heating rate). The main mass-loss stage occurs at approximately 350–470 °C. With the increase in the heating rate, the mean DTG peaks shift to a higher temperature: from 387.1 ± 10.3 °C at 2 °C/min to 427.9 ± 15.2 °C at 20 °C/min. The kinetics of thermal decomposition were investigated using isoconversional analysis methods. The apparent activation energy of the main mass-loss peak calculated by the Kissinger method was 200.85 kJ/mol (R^2^ = 0.99). Gas-phase mass-spectrometric analysis tentatively assigned signals to water, CO, CO_2_, and acrylate-derived fragments of organic chains. A thermal analysis-guided debinding schedule was developed and experimentally demonstrated, resulting in the production of bulk ceramics with measurable optical translucence (the full transmittance and in-line transmittance were 54.5% and 12%, respectively, for a sample 1.2 mm thick). The absence of large cracks and delamination was observed in both green and brown bodies by scanning electron microscopy.

## 1. Introduction

Stereolithographic 3D printing (stereolithography, SLA) is a widely used method of additive manufacturing, allowing the fabrication of high-precision ceramic components. This enables the realization of complex geometries that remain unachievable through traditional processing methods [1,2]. The method is based on the layer-by-layer photopolymerization of highly filled ceramic suspensions, where an organic monomer serves as the liquid phase for inorganic powders, followed by a multi-stage post-processing sequence involving organic binder removal (debinding) and high-temperature sintering. Among the various acrylate monomers applied in this area, 1,6-hexanediol diacrylate (HDDA) appears to be the most commonly used due to its exceptionally low dynamic viscosity (5–12 mPa·s) and rapid polymerization kinetics [3,4,5,6,7,8,9,10], as well as resin systems based on it [11,12,13,14,15].

Despite the widespread application of HDDA-based systems, a significant portion of current research focuses on complex multi-component mixtures incorporating various monomers or oligomers, such as isobornyl acrylate (IBOA), trimethylolpropane triacrylate (TMPTA) or polyethylene glycol diacrylate (PEGDA) [8,10,11], which obscure the specific thermophysical contributions of the HDDA-based matrix and complicates the interpretation of mass-loss data. Furthermore, the critical influence of heating rates and gas atmospheres on the thermal decomposition process is frequently overlooked, even though these parameters directly influence the evolution of internal pressure gradients and the subsequent formation of defects and delamination [12].

The challenges of binder removal are particularly acute in the fabrication of massive, bulk ceramic objects, where heat and mass transfer limitations are magnified. In this case, debinding typically requires a carefully controlled thermal treatment in an inert (Ar, N_2_) or reducing (Ar + H_2_) atmosphere to avoid uncontrolled oxidation and manage the kinetics of volatile release. For example, in [16], these effects were confirmed by thermal analysis and neutron imaging in the case of vacuum and argon. Another complication is the necessity of heating schedules lasting more than 12 h for the same reasons. The treatment time can be reduced for thin-walled objects, but it still requires careful selection of the temperature profile near the main mass-loss peaks [17,18,19]. For optical materials such as cerium-activated complex multi-component garnets, maintaining structural integrity during this phase is crucial for achieving the desired optical and functional properties in the sintered ceramics. For example, (Gd,Y)_2_Al_2_Ga_3_O_12_:Ce (GYAGG:Ce) is highly valued for scintillation and luminescent properties [20]. The inherent anisotropy of 3D-printed parts, characterized by weaker inter-layer adhesion, further enhances the risk of failure at the interfaces where volatile decomposition products tend to accumulate [7].

The literature contains previously reported studies on the thermal degradation of polymers and green bodies based on HDDA [21,22,23]. However, these studies do not consider the thermal decomposition of highly filled green bodies using the vat polymerization method. A high concentration of ceramics can alter the composition of the organic phase, as well as the conditions of heat and mass transfer during debinding. These factors are especially important when working with bulk and geometrically complex ceramic parts, where the rapid release of volatile products can lead to the formation of internal pressure gradients and cause cracking and delamination. A comprehensive analysis of the kinetics of decomposition using non-isothermal methods at various heating rates using Kissinger, Kissinger–Akahira–Sunose (KAS), and Flynn–Wall–Ozawa (FWO) models makes it possible to calculate the effective kinetic parameters. This provides a basis for the rational design of optimal temperature conditions that ensure a constant and controlled rate of mass loss, which minimizes pressure gradients inside the material.

The study of the internal structure of green bodies based on pure HDDA allows one to identify the structural features formed during photopolymerization, which may act as stress concentrators and initiate cracking and delamination at the debinding stage [8].

Thus, the novelty of this work lies not in the DLP printing technology itself, but in the systematic thermal and kinetic analysis data of the highly loaded GYAGG:Ce/HDDA green bodies, as well as in the comparison of the obtained thermal analysis data with the experimentally demonstrated procedure for removing the binder from bulk and complex-shaped ceramic structures.

In this regard, the aim of this work was to characterize the non-isothermal thermal decomposition curve and the apparent kinetics of the GYAGG:Ce/HDDA green body obtained using the DLP printing method; to evaluate the effect of the heating rate and atmosphere during processing; and to use the obtained thermal analysis data to develop and experimentally demonstrate a controlled regime for the removal of the binder from bulk and geometrically complex GYAGG:Ce ceramics.

## 2. Materials and Methods

In this work, 1,6-hexanediol diacrylate (HDDA, 80%, contains 100 ppm of methoxyphenol as an inhibitor, Sigma-Aldrich, St. Louis, MO, USA) was used as the acrylate monomer. Its dynamic viscosity was 5.99 mPa·s at 25.0 °C, the refractive index was n_20_ = 1.458, and the density was ρ = 1.019 g/cm^3^. The commercial monomer was verified by IR spectroscopy (Vertex 70, Bruker, Bremen, Germany; the FTIR spectra are presented in Figure S1). To reduce the viscosity and ensure steric stabilization of the powder particles, a hyperbranched polyester dispersing additive, DisperBYK (BYK-Chemie GmbH, Wesel, Germany), was introduced into the suspension at 2 mg/m^2^ of the powder surface area. Diphenyl(2,4,6-trimethylbenzoyl) phosphine oxide (TPO, American Barki Chemical Inc, Norwalk, CT, USA) was added as a photoinitiator at 1 wt.% relative to the monomer mass.

The ceramic filler was a powder of a complex oxide with a garnet structure of the composition Gd_1.194_Y_1.791_Ce_0.015_Al_2_Ga_3_O_12_ (GYAGG:Ce), synthesized by co-precipitation with ammonium bicarbonate from nitric acid solutions of cations of the corresponding metals. The precipitate formed as a result of synthesis was subjected to heat treatment at 1250 °C for 2 h (LHT 04/17, Nabertherm, Lilienthal, Germany) followed by grinding in a ball mill. Laser diffraction measurements were performed using a MasterSizer 2000 (Malvern, Malvern, PA, USA), yielding a median particle size (d_50_) of 2.6 μm (Figure S2a). The specific surface area of the powder (6.54 m^2^/g) and the pore volume (0.008 cm^3^/g) were determined by low-temperature nitrogen adsorption on a NOVA 4200e instrument (Quantachrome, Boynton Beach, FL, USA). According to the scanning electron microscopy (SEM) images obtained using JSM 7100 F (Jeol, Tokyo, Japan), the initial powder particles had a size of 200–300 nm and had a rounded shape with a smooth surface (Figure S2b). The phase composition of the powder was studied by X-ray diffraction analysis using a desktop diffractometer D2 Phaser (Bruker, Billerica, MA, USA) equipped with a CuKα_1.2_ X-ray tube. The obtained diffraction patterns were consistent with the cubic garnet structure *Ia-3d* (S.G. 230), and no signals indicating the presence of impurity phases were detected within the sensitivity range of the device (Figure S3).

The photopolymerizable suspension was prepared by sequentially adding the oxide powder to a premixed organic base (mixture of monomer, dispersant and photoinitiator) with constant stirring until a solid loading of 35 vol.% (76 wt.%) was obtained. This composition was selected as a compromise between sufficient green body density and maintaining a low viscosity of the suspension, which directly affects the printability.

Green bodies of complex shape were manufactured using an open architecture desktop 3D printer Ember (Autodesk, San Francisco, CA, USA) with λ = 405 nm and W = 15.0 mW/cm^2^ [14]. A monolithic disk-shaped green body (d = 20 mm, h = 2 mm) was printed using a Photon Ultra 3D printer (Anycubic, Shenzhen, China) with λ = 405 nm and W = 2.35 mW/cm^2^. The thickness of the printing layer was 50 μm. The exposure dose for a single layer was approximately the same in all cases and amounted to 80 mJ/cm^2^. Immediately after 3D printing, the green bodies were washed several times in fresh HDDA monomer in a laboratory ultrasonic bath and then post-cured in a UV chamber. The same green body composition and washing/post-curing procedure were used for the thermal analysis and debinding experiments.

Differential scanning calorimetry (DSC) was performed on a NETZSCH DSC 204F1 Phoenix device (Netzsch-Gerätebau GmbH, Selb, Germany) at a heating rate of 20 °C/min in an argon atmosphere (flow rate of 60 mL/min). The measurements were carried out in aluminum crucibles; the mass of the samples was ~3–5 mg.

Thermogravimetric analysis (TGA) was performed on a NETZSCH TG 209F1 Iris instrument equipped with a QMS 403D Aëolos mass-spectrometric device (Netzsch-Gerätebau GmbH, Germany) at heating rates of 2, 4, 8 and 20 °C/min. The main kinetic measurements were performed under argon at a gas flow rate of 50 mL/min. The experiments were performed in Al_2_O_3_ crucibles in an atmosphere of argon and dry air at a gas flow rate of 50 mL/min. The weight of the sample was 50 ± 0.5 mg. A separate comparative thermogravimetric experiment was conducted using dry air, pure argon, and an Ar/H_2_ mixture using an SDT Q600 instrument (TA Instruments, New Castle, DE, USA) in the temperature range from 25 to 500 °C with a constant heating rate of 2.5 °C/min. These data were used to assess the qualitative effect of the atmosphere and were not taken into account when conducting the main multi-stage kinetic analysis.

Before measuring, an empty curve (empty crucible) was recorded for each combination of heating rate and gas atmosphere under identical conditions, after which baseline subtraction was applied to all experimental data.

Evolved gas analysis was performed using a Netzsch Aeolos QMS 403D (Selb, Germany) quadrupole mass spectrometer coupled directly to the thermal analyzer. The instrument was operated in multiple ion detection (MID) mode with simultaneous monitoring of five ion channels assigned to *m*/*z* = 15, 18, 28, 39, and 44 (corresponding to CH_3_^+^, H_2_O^+^, CO^+^/C_2_H_4_^+^, C_3_H_3_^+^, and CO_2_^+^, respectively). All channels used a Channeltron (CH-TRON) secondary-electron-multiplier detector with an applied SEM voltage of 1200 V. The dwell time was 1 s per channel; mass resolution was set to 25 for *m*/*z* = 15 and 50 for all remaining channels. The ion source was a high-sensitivity yttria-coated filament (HS Yttria, Fil 2, SET 0) operated at an emission current of 0.80 mA and an emission-protection threshold of 3.50 A. The ion-source voltages were: ion reference 110 V, cathode 70 V, focus 10 V, field axis 10 V, and extraction 110 V; RF polarity was set to normal. Mass scale correction was enabled; zero-gas subtraction was not applied.

All samples for thermal analysis were weighed on a SARTORIUS ME235P analytical balance (Sartorius AG, Göttingen, Germany) with an accuracy of 10^−5^ g.

To ensure the statistical robustness and reproducibility of the kinetic parameters, four independent TGA/DTG measurements (n = 4) were performed for each heating rate. The TG curves were normalized to the initial sample mass. To obtain the mean TG and DTG profiles, the four curves recorded at each heating rate were linearly interpolated onto a common temperature grid. The data on mass loss obtained using a thermogravimetric analyzer were additionally compared with independent gravimetric measurements taken before and after processing using analytical scales (Table 1) to verify the reliability of the data on the removal of the organic fraction.

Conversion degree α is defined as the ratio of the instantaneous mass loss at a specific temperature to the total mass loss observed at the end of the analyzed thermal decomposition stage (1):

(1) $$\alpha \left(T\right)=\frac{m_{s}\;-\;m(T)}{m_{s}{\;-\;m}_{f}}$$

where m_s_ is the sample mass at the beginning of the analyzed decomposition stage, m(T) is the mass at temperature T, and m_f_ is the final mass. Thus, α represents the conversion of the organic fraction during thermal decomposition and should not be interpreted as the polymerization conversion of HDDA during printing.

The kinetics of thermal decomposition of the green bodies were evaluated using the Kissinger method (2) based on the analysis of temperature peaks of the maximum decomposition rate at various heating rates according to the following equation [24]:

(2) $$\left(\frac{\beta }{T_{p}^{2}}\right)=ln\left(\frac{AR}{E_{a}}\right)-\;\frac{E_{a}}{RT_{p}},$$

where β is the heating rate, T_p_ is the absolute DTG peak temperature, E_a_ is the activation energy, A is the pre-exponential factor, and R is the universal gas constant.

The Kissinger–Akahira–Sunose (KAS) and Flynn–Wall–Ozawa (FWO) isoconversional methods allow the estimation of the activation energy using the Arrhenius integral equation for fixed degrees of conversion α, which allows a more accurate description of the kinetics of multi-stage decomposition processes. The KAS Equation (3) has the following form [25]:

(3) $$ln\left(\frac{\beta }{T_{\alpha }^{2}}\right)=ln\left[\frac{AR}{E_{\alpha }g(\alpha )}\right]-\;\frac{E_{\alpha }}{RT_{\alpha }},$$

where T_α_ is the absolute temperature corresponding to conversion degree α (α = 0.1; 0.2…), E_α_ is the apparent activation energy at that conversion and g(α) is the integral conversion function.

At the same time, the FWO Equation (4) uses a logarithmic approximation that simplifies data plotting, although it is somewhat less accurate for nonlinear curves, and can be written as follows [26]:


(4) $${log}_{10}\beta \;=\;{log}_{10}\left[\frac{AE_{\alpha }}{Rg(\alpha )}\right]-2.315-0.4567\frac{E_{\alpha }}{RT_{\alpha }}.$$

The kinetic parameters obtained using these methods are considered to be apparent activation energies characteristic of the composite under study and the experimental conditions. Since the starting material is a highly loaded multi-component system, the calculated values should not be interpreted as the intrinsic activation energies of pure HDDA or as direct evidence of the existence of a unique decomposition mechanism.

This scientifically rigorous approach provides the necessary theoretical foundation for the rational design of optimized temperature–time profiles, ensuring a constant and controlled rate of mass loss to minimize internal pressure gradients and mitigate the formation of structural defects in bulk ceramic components.

The debinding of bulk and complex-shaped green bodies was carried out by heating to 500 °C in a horizontal tubular furnace with a cylindrical quartz reactor. The volume of the quartz reactor used for the burnout is approximately 400 mL. The process was carried out in a flowing gas mixture of 95% Ar + 5% H_2_ at a gas flow rate of 50 cm^3^/min. The main multi-speed kinetic measurements were carried out in a pure argon environment; therefore, the kinetic parameters obtained during these experiments were used as a reference for thermal analysis when selecting the temperature range and heating rate, rather than as a quantitative kinetic model for the binder removal process in a 95% Ar + 5% H_2_ environment.

Based on the TG and DTG results, the debinding schedule was chosen in such a way as to reduce the instantaneous rate of formation of volatile products in the main mass-loss region. The first step included heating from room temperature to 300 °C at 1.0 °C/min. At the second step, it was reduced to 0.4 °C/min between 300 and 450 °C, encompassing the main mass-loss peak as well as high-temperature shoulder, observed on the DTG curve at slow heating rates. After an isothermal hold at 450 °C for 1 h, the green bodies were heated to 500 °C at 1.0 °C/min followed by an additional hold for 0.5 h. Finally, the resulting brown bodies were placed into a muffle furnace and the residual carbon was oxidized in air at 500 °C for several hours.

This graph was experimentally demonstrated using a disk with a thickness of 2 mm and products of complex shapes. It does not claim to be a universal optimized graph for removing the binder from parts of arbitrary dimensions or wall thicknesses. However, it successfully demonstrated its viability for the specified range of objects.

Sintering of the ceramics was carried out in an oxygen atmosphere by heating to 1720 °C with an isothermal exposure of 2 h. Sintering in an oxygen atmosphere is used to achieve high density and excellent performance characteristics by suppressing the evaporation of elements, eliminating pores due to the rapid diffusion of gases, and preventing defects associated with a reduction in the material’s volume. The total and linear transmittance of the sample in the wavelength range from 400 to 700 nm was evaluated using a Specord Plus spectrophotometer (AnalytikJena, Jena, Germany) with an integrating sphere.

The study of the internal microstructure of both green bodies and intermediates after debinding (“brown body”) was performed using the SEM method. The samples were embedded in epoxy resin and polished in cross-section on a P12M grinding and polishing machine (Polylab, Moscow, Russia) with an automatic holder using abrasive wheels based on silicon carbide of different grain sizes (from P180 to P2500). The final polishing was carried out using Aquapol-M diamond suspensions (Kemet, Kent, UK) with particle sizes of 0.5, 0.25, and 0.1 μm applied to short-nap polishing cloths.

## 3. Results and Discussion

**Figure 1:**
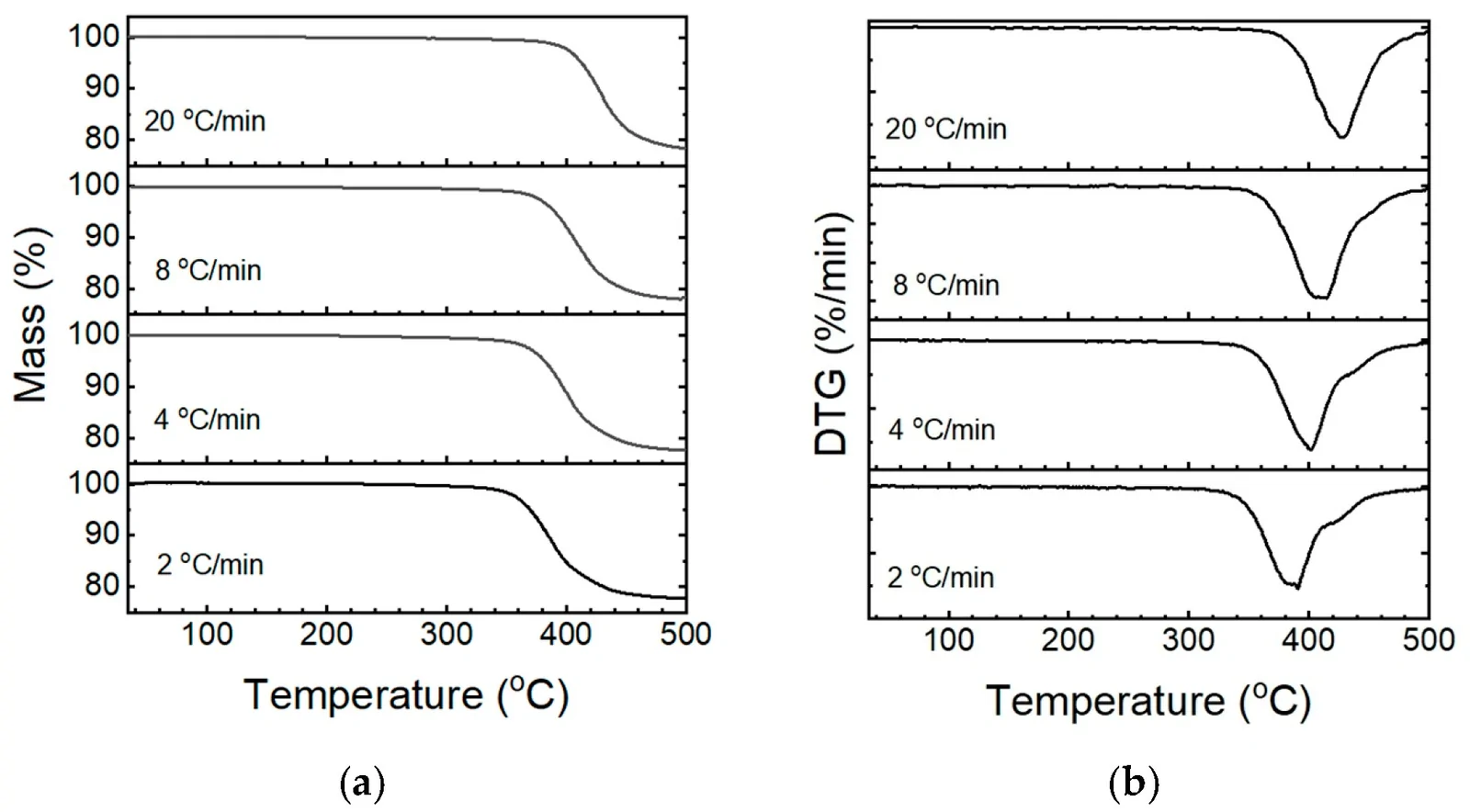
Mean TGA (**a**) and DTG (**b**) curves of the GYAGG:Ce/HDDA green bodies recorded under argon at heating rates of 2, 4, 8 and 20 °C/min. Each curve represents the mean of four independent measurements. The DTG curves are shown with individually scaled *Y*-axes for easier comparison of peak positions and curve shapes.

Thermogravimetric curves obtained at various heating rates in the range of 2 to 20 °C/min (Figure 1a) demonstrate that the main mass loss occurs at approximately 350–470 °C and corresponds to the predominant thermal decomposition of the organic HDDA-containing matrix. With the increasing heating rate, the temperature corresponding to the maximum mass-loss rate (DTG peak) is shifted towards the higher values (see Table 2). This shift is a well-known feature of non-isothermal thermogravimetric analysis and is primarily due to kinetic factors. At higher heating rates, the system has less time to achieve a given conversion degree at lower temperatures, so higher temperatures are required to achieve the same reaction rate. Additionally, the observed shift may be related to the thermal lag between the furnace and the sample, as well as internal limitations in heat and mass transfer. As a result, the apparent decomposition temperatures increase with the increasing heating rate.

The total mass loss is approximately 22–23%, and the residue predominantly corresponds to the inorganic GYAGG:Ce phase. Since the calculated mass fraction of the ceramic powder is close to this value, it suggests that the organic component is completely or almost completely removed by 500 °C.

All DTG curves are characterized by one dominant negative peak corresponding to the maximum rate of mass loss during decomposition of the organic matrix (Figure 1b). The DTG peak becomes sharper, and its apparent amplitude increases with an increase in the heating rate. The DTG curves in Figure 1b are shown on the Y-axis on an individual scale for clarity, so their absolute maxima should not be compared directly. As the heating rate increases, the minimum position consistently shifts to higher temperatures (Table 2). Values are presented as mean ± standard deviation, n = 4.

The height of the negative peak also increases significantly with a higher heating rate. Since the DTG data are presented in %/min, an increase in the absolute value of the mass-loss rate is a natural consequence of faster heating. In addition, the panels have different scales of the Y-axis, so it is impossible to visually compare the amplitudes between them directly.

On the high-temperature side of the main peak, especially at 2–8 °C/min, there is a shoulder or change in slope in the range of about 420–460 °C. This can be an overlap of at least two processes. These can be the sequential destruction of various fragments of the cross-linked polymer network, the decomposition of more heat-resistant primary degradation products, or processes limited to the removal of volatile products through a highly filled ceramic structure. As the heating rate increases, the individual contributions overlap more strongly, as a result of which the peak becomes wider and less resolved.

The obtained results are in a good agreement with the literature: decomposition begins at about 300 °C, the peak activity is observed at about 400 °C [27], and the process is significantly influenced by the atmosphere and the rate of heating [28].

At a heating rate of 2.5 °C/min, thermogravimetric curves and their first derivative were also obtained for composites of a similar composition under different atmospheres—air, argon, and a mixture of argon with 50% hydrogen (Figure S4). It is shown that the atmosphere of the process has a noticeable effect on the position of the DTG maxima and the amount of mass loss. In air, the main DTG maximum was observed at 323 °C, whereas in inert media (Ar and Ar + H_2_) it shifts to higher temperatures (~380 °C), which can be explained by the absence of oxidative processes [16]. In addition, for the same reason, the secondary peak associated with the oxidation of thermal decomposition products of impurities in HDDA is most pronounced in air. The mass loss was about 20% in air, 23% in argon, and only 15% in the Ar + H_2_ mixture. The differences are probably due to the nature of the reactions taking place—in air, the carbon residue mostly burns out due to the presence of additional oxidative processes at temperatures above 400 °C, and more stable carbon-containing compounds are formed in the reducing medium Ar + H_2_, thus reducing mass loss, whereas in argon, the removal of volatile decomposition products occurs most actively [29].

These data on the comparison of atmospheres were obtained using pure argon and a mixture of argon and hydrogen in a 1:1 ratio, so they should not be considered as a direct kinetic description of an atmosphere consisting of 95% argon and 5% hydrogen, which is used in the binder removal process. The latter atmosphere was chosen as the experimentally studied processing condition, while the presented comparison primarily demonstrates the dependence of the decomposition process on the gas composition.

The instrument-derived and gravimetrically measured mass losses were in close agreement, with mean differences not exceeding 1.70 percentage points (Table 1). This comparison was used to independently verify the consistency of the mass-loss values obtained through TGA, rather than to independently verify the decomposition kinetics.

**Figure 2:**
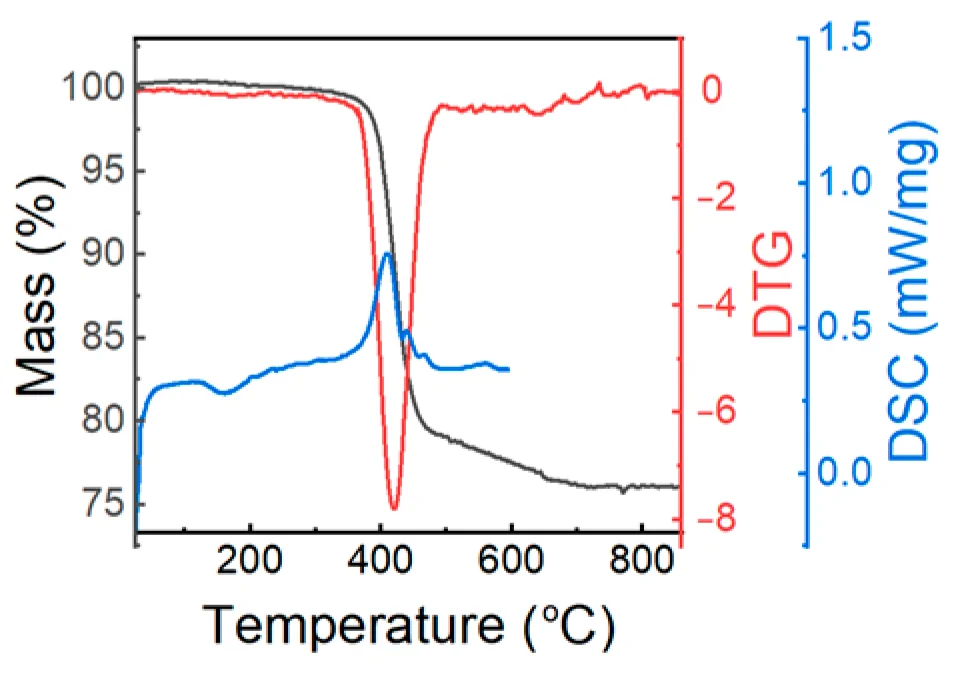
TGA, DTG and DSC curves obtained in an inert atmosphere at a heating rate of 20 °C/min.

For a more detailed study of the thermal effects occurring at a heating rate of 20 °C/min, along with thermogravimetric analysis and the first derivative, differential scanning calorimetry was also performed (Figure 2).

The curves indicate a multi-stage thermal decomposition of composites. At low temperatures (below ~300 °C), there is only a slight loss of mass (~1–2%), which can be explained by the removal of moisture and residual low-molecular-weight compounds. This stage is accompanied by a weak thermal effect in the DSC signal. The main decomposition occurs in the temperature range 350–450 °C and is characterized by a sharp weight loss of about 24%, which corresponds to the destruction of the organic polymer matrix. The DTG curve demonstrates a pronounced peak with a maximum at a temperature of ~400–420 °C. It is noteworthy that the asymmetric shape of the DTG peak, including the visible protrusion, indicates the presence of overlapping decomposition processes rather than a simultaneous reaction. This behavior may be due to the possible influence of additives or residual forms of the monomer. In the intermediate-temperature range (450–700 °C), there is a gradual loss of mass, which is probably associated with the removal of more thermally stable decomposition products and the oxidation or decomposition of residual carbon-containing compounds. The corresponding DTG signal is weak and dilated, indicating a diffusion-limited or kinetically slow process. At temperatures above ~700 °C, the TG curve reaches a plateau, which indicates the completion of the decomposition process and the formation of a stable inorganic residue corresponding to the ceramic phase. Minor disturbances observed at higher temperatures are due to exposure to instruments and/or changes in the gaseous atmosphere, rather than internal transformations of the material.

**Figure 3:**
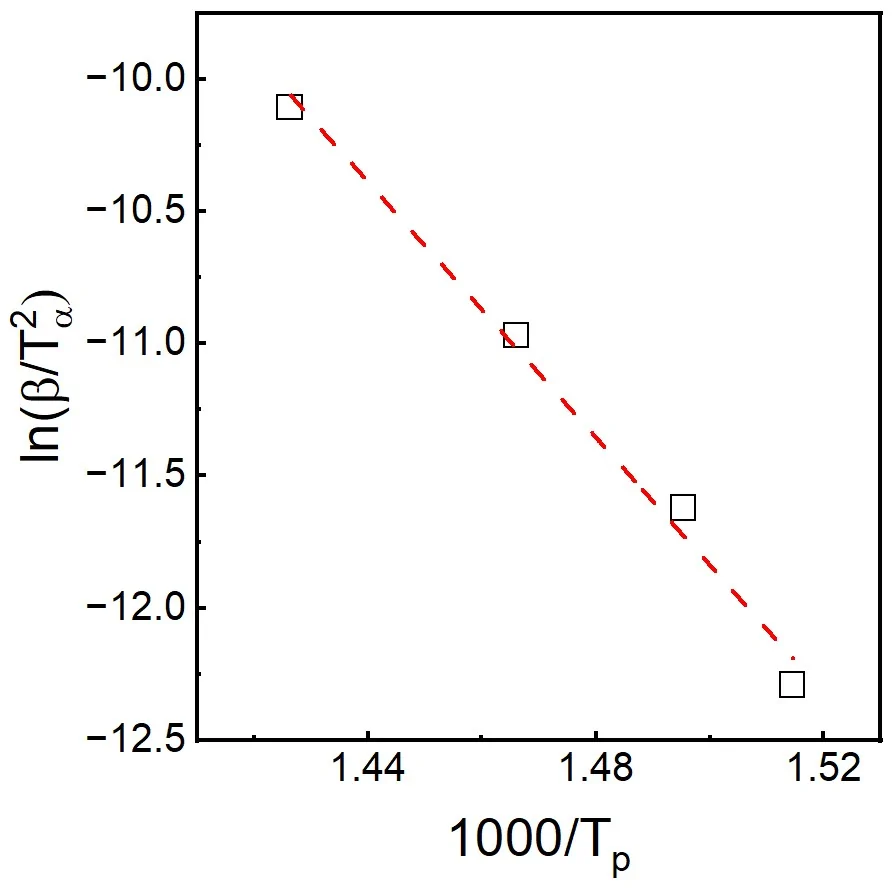
Kissinger plot of ln(β/T^2^) versus 1000/T for the principal decomposition stage.

Figure 3 shows the dependence corresponding to the Kissinger equation and plotted from the temperatures of the maxima of the DTG curves at different heating rates; that is, each point corresponds to the temperature of the main peak of mass loss.

The graph represents a negative linear relationship confirming a systematic shift in the temperature of the DTG peak. It depends on the heating rate that was used to estimate the apparent activation energy of the dominant decomposition stage. The activation energy calculated from the slope of the curve was E_a_ = 200.85 kJ/mol, and the coefficient of determination R^2^ was 0.99. The observed deviations of individual points from the regression line are consistent with the asymmetric DTG profiles and the overlap of several decomposition processes. The obtained E_a_ value is close to the results described in [22]—the activation energy of a pure HDDA-based polymer according to the Kissinger method was 170 kJ/mol. The slightly higher value in the case of our work may be explained by the presence of a ceramic filler in the polymer matrix, which increases the thermal stability of the system under study. A similar effect was described in [30], where the addition of a binding agent to the polymer led to an increase in the activation energy.

**Figure 4:**
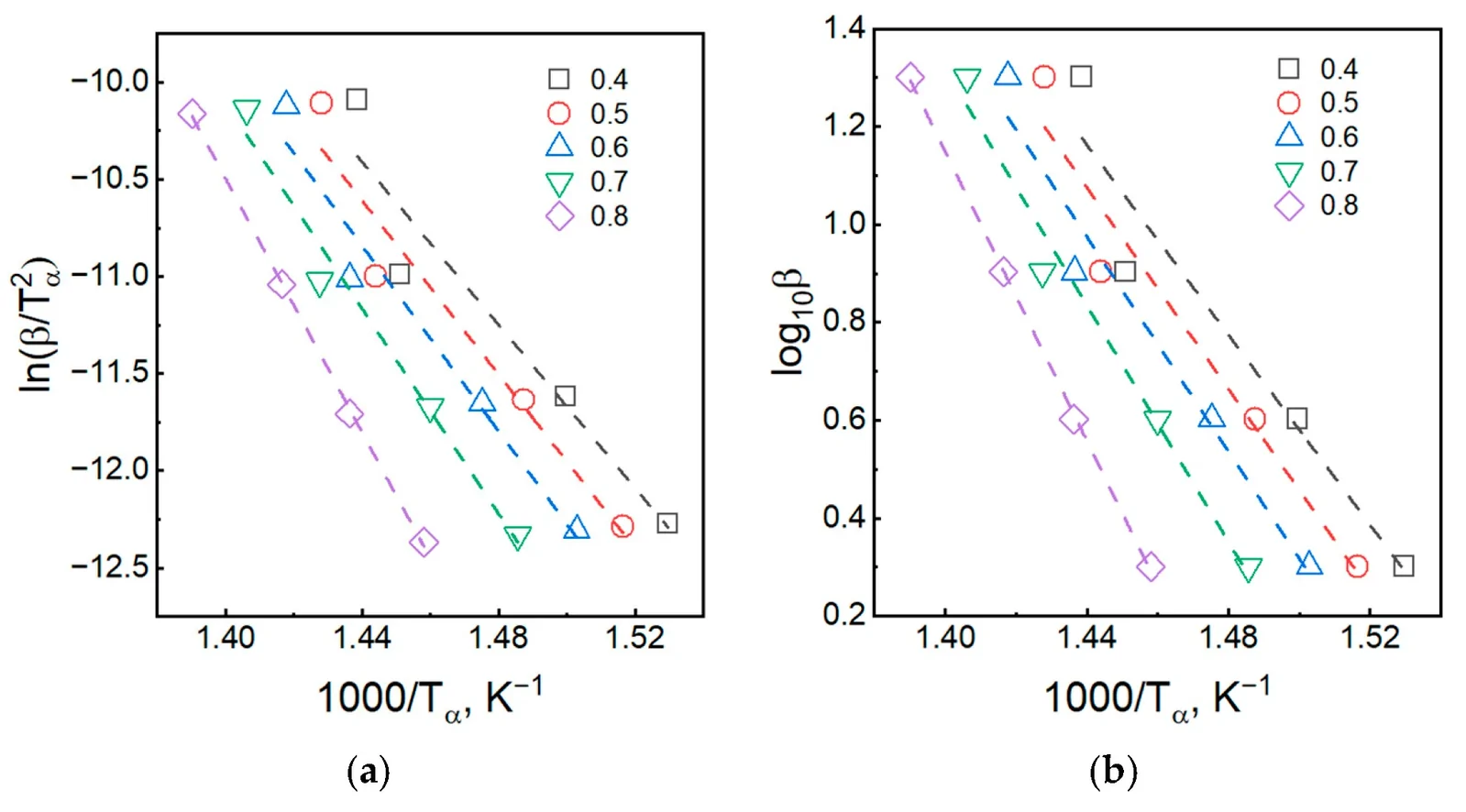
Isoconversional regression plots obtained using the (**a**) KAS and (**b**) FWO methods at selected conversion degrees.

Figure 4 shows the results of the isoconversional analysis using the Kissinger–Akahira–Sunose (Figure 4a) and Flynn–Wall–Ozawa (Figure 4b) methods.

The KAS graphs show a gradual increase in the absolute slope depending on the degree of transformation, which indicates an increase in the apparent activation energy during decomposition. The isoconversion lines also shift towards lower values of 1000/T_a_.

The FWO analysis gives results similar to those obtained by the KAS method. The absolute slopes of the curves increase with the increasing degree of conversion, which is consistent with a gradual increase in the apparent activation energy. The close correspondence of the results indicates that the observed dependence on the degree of conversion is not solely related to the use of a single mathematical approximation. Nevertheless, regressions for low degrees of transformation remain less linear than those obtained in the central part of the decomposition process.

The values increase as α increases, reflecting the temperature increase required to achieve a high degree of conversion. The quality of the linear approximation significantly improves in the range from medium to high α (0.6–0.8), while the data for low α (0.1–0.2) show significant deviations from linearity.

**Figure 5:**
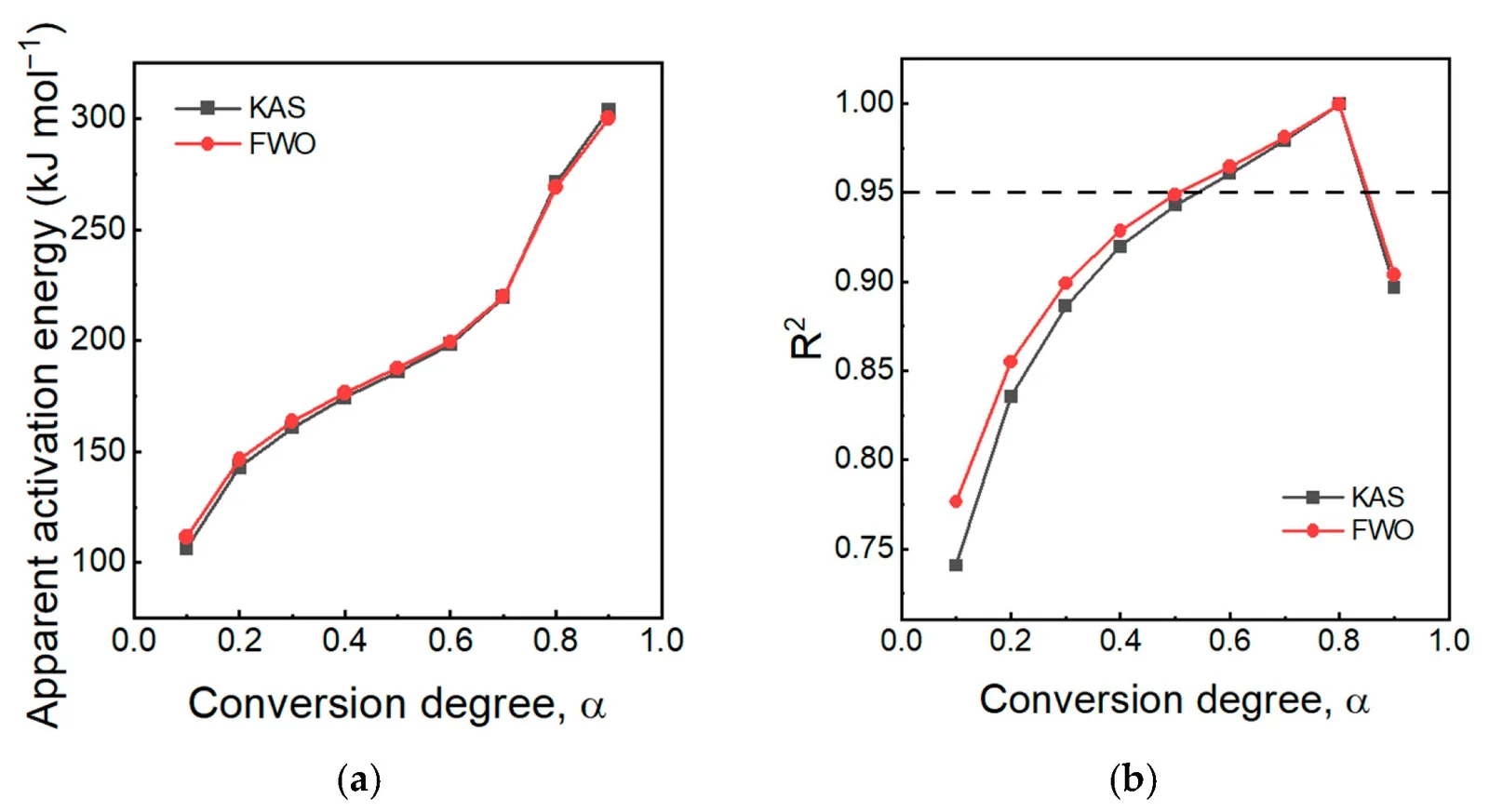
(**a**) Apparent activation energy E_a_ and (**b**) coefficients of determination R^2^, as functions of the conversion degree α, for the KAS and FWO models. The horizontal dashed line in panel (**b**) indicates R^2^ = 0.95. The E_a_ values were calculated from the mean TGA profiles obtained at each heating rate.

The apparent activation energies obtained by the KAS and FWO methods (Figure 5a) are in good agreement in the range where linear regressions yield reliable results.

Since only four heating rate values were available for each isoconversional regression, the uncertainty of the obtained activation energy values was estimated using regression statistics, rather than the standard deviation of the four peak temperatures obtained by differential thermogravimetry. For the kinetic analysis, the mean TG/DTG profile obtained for each heating rate was used to determine the characteristic temperatures at each conversion degree. Therefore, a single kinetic dataset was generated for each heating rate from the averaged measurements, and the resulting apparent activation energies were calculated once for each conversion degree. The standard deviations of the replicate DTG measurements were therefore not used as uncertainties of the calculated activation energies.

Both methods exhibit a noticeable increase in E_a_, from about 105–110 kJ/mol at low α to about 300 kJ/mol at α = 0.8. Such a strong dependence of apparent activation energy on the degree of conversion is consistent with the complex and multi-stage nature of the degradation process. Figure 5b shows that the most reliable linear-regression range is approximately α = 0.6–0.8. The increase in E_a_ may reflect the sequential decomposition of different components and degradation products of the cross-linked structure and the growing influence of limitations associated with mass transfer in a highly filled ceramic composite.

The Kissinger activation energy of 200.85 kJ/mol is higher than the values obtained for some polymer systems containing HDDA. However, direct comparison should be approached with caution, since in this case the measurements were carried out on a photopolymerized GYAGG:Ce/HDDA composite with a high filler content, rather than on an isolated polymer. The higher value may be due to differences in composition and cross-linking, as well as heat and mass transfer within the ceramic filler. The data obtained do not allow for a quantitative separation of these factors.

A similar course of the curves corresponding to the KAS and FWO methods was observed in systems based on poly(2-ethyl hexylacrylate) [31], the decomposition of which was also a multi-stage process: a pronounced main peak in mass loss was accompanied by a second minor peak at a higher temperature.

**Figure 6:**
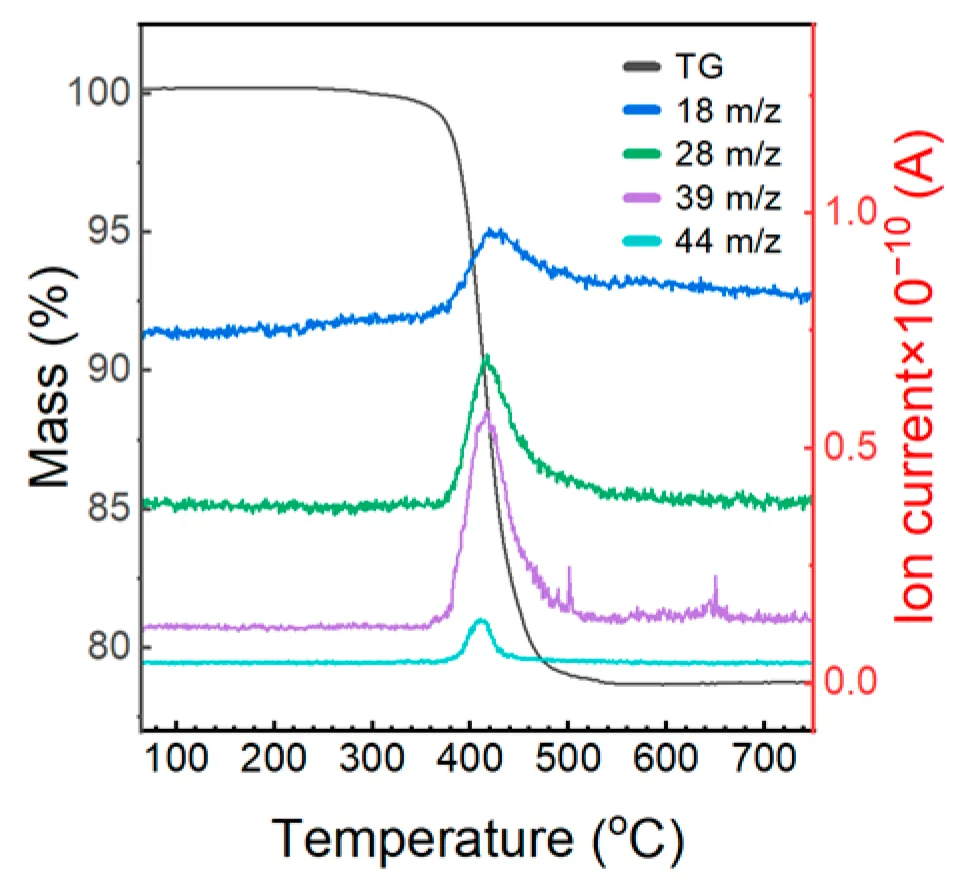
TG curve and selected mass-spectrometric signals recorded during thermal decomposition of GYAGG:Ce/HDDA green body under argon atmosphere.

The analysis of the gas phase formed during the thermal decomposition of the green body was carried out using mass spectrometry (Figure 6). The signals were tentatively attributed to water vapor (18 *m*/*z*), a signal corresponding to CO (28 *m*/*z*), fragments of carbon chains formed during the rupture of acrylate groups, for example, the C_3_H_3_^+^ cation (39 *m*/*z*), and a signal consistent with CO_2_ (44 *m*/*z*). Since, during electron impact ionization, individual *m*/*z* values are not unique to any single type of molecule, these assumptions are considered tentative rather than definitive. There are no studies in the literature devoted to the composition of exhaust gases produced during the thermal decomposition of polymers based on the presented GYAGG:Ce/HDDA composite; however, similar compounds are detected during the analysis of other acrylates and their copolymers [32].

FTIR spectroscopic analysis of resinous condensates collected in the cold zone of the quartz reactor showed qualitatively similar spectra after pyrolysis in vacuum, Ar and Ar + 5% H_2_ (Figure S5). The spectra were dominated by bands associated with aliphatic C-H (2850–3000 cm^−1^), carbonyl (1720–1730 cm^−1^), and C-O-containing groups (1050–1300 cm^−1^), indicating the formation of condensed oxygen-containing decay products rather than simple evaporation of unchanged HDDA. Since the starting material was photopolymerized and the condensates were collected after thermal decomposition, the spectra were interpreted as the spectra of the decomposition products of the cured organic phase, rather than as the spectra of unmodified HDDA. A library search yielded moderate correlation values (approximately 68–70%) and mostly coincided with long-chain aliphatic aldehydes, ester-containing compounds, and resinous materials (Figure S6).

The condensates demonstrate very similar sets of library correspondences, while the condensate obtained in vacuum showed a slightly stronger correspondence with aliphatic aldehydes. Since condensates are multi-component mixtures, and library correspondences are not specific enough for molecular identification, these results were interpreted only in terms of the main classes of compounds. Thus, the results of FT-IR spectroscopy provide qualitative information about the composition of functional groups and the dominant classes of condensates, but they do not allow for the unambiguous identification of molecules. In general, it can be noted that the obtained resinous condensates are moderately soluble in isopropyl alcohol, and are highly soluble in acetone and the original monomer (HDDA).

**Figure 7:**
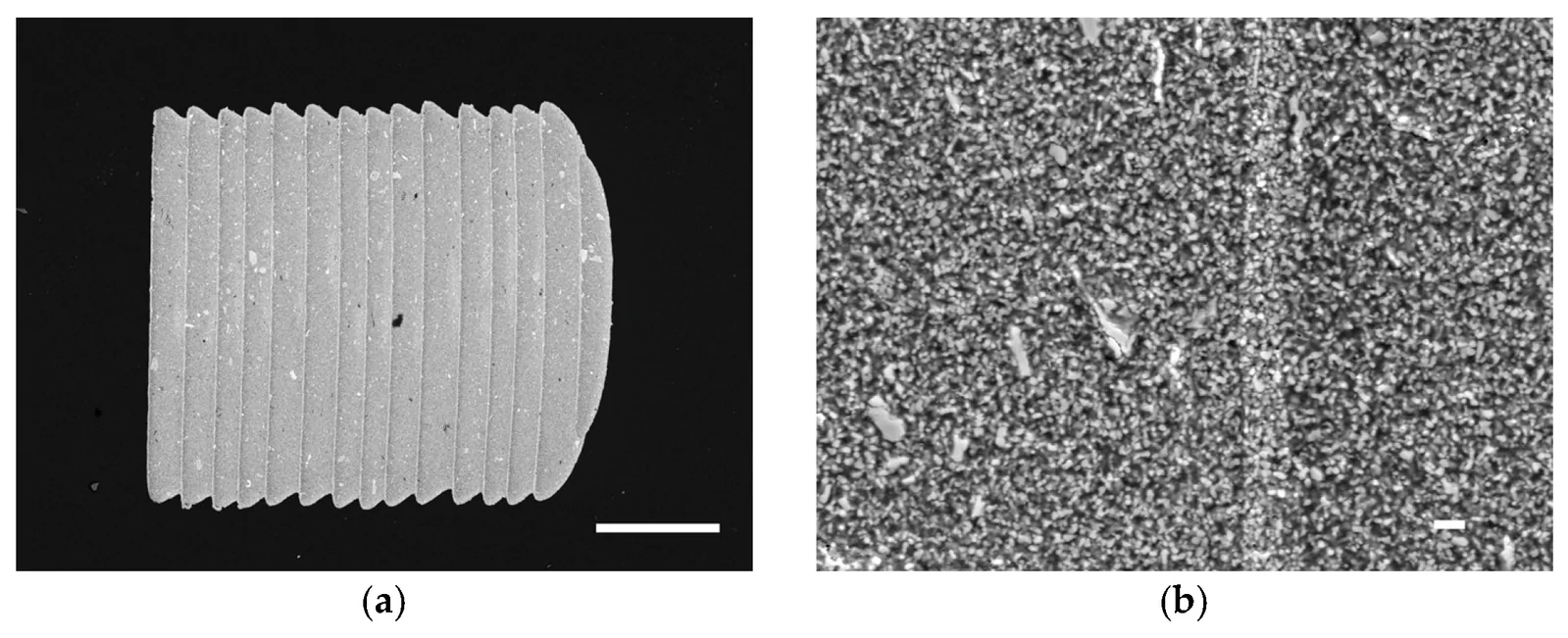
SEM images of a polished cross-section of the GYAGG:Ce/HDDA green body at magnifications of (**a**) 200× and (**b**) 5000×. The scale bars correspond to 100 and 1 μm, respectively.

**Figure 8:**
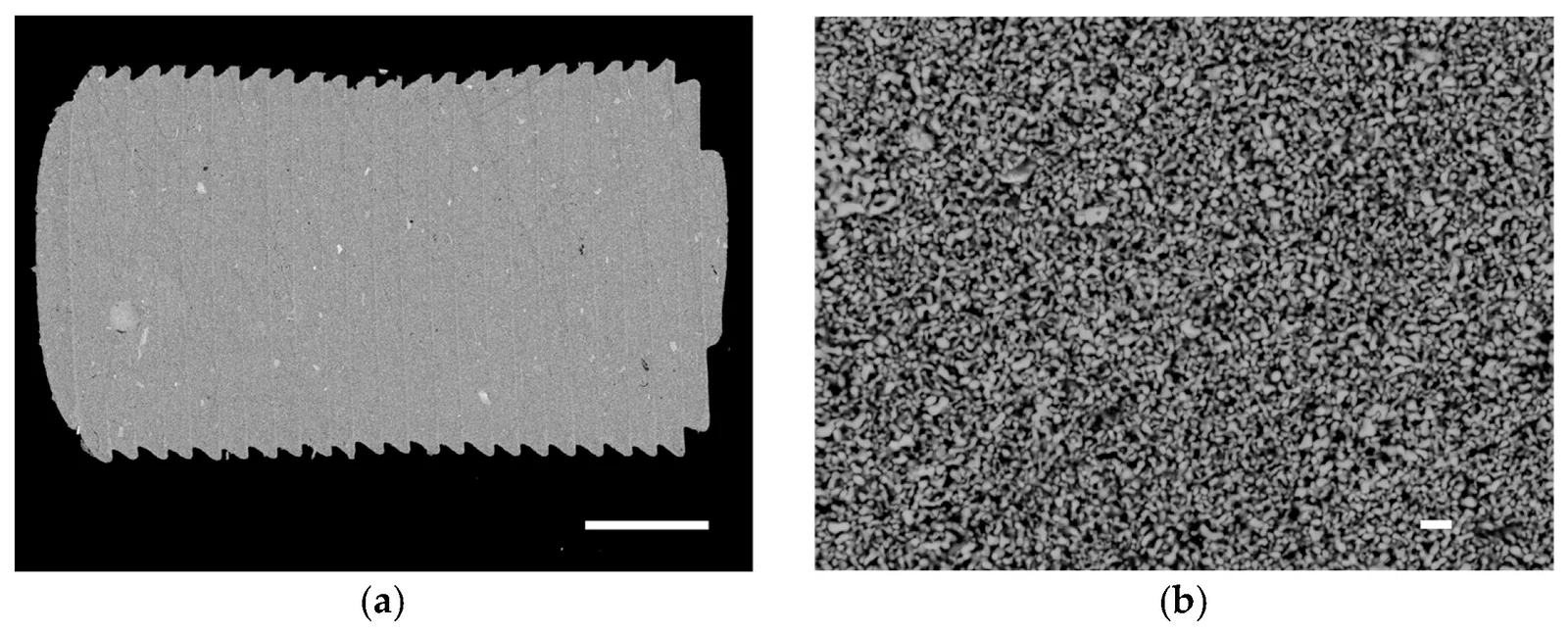
SEM images of a polished cross-section of the GYAGG:Ce brown body at magnifications of (**a**) 200× and (**b**) 5000×. The scale bars correspond to 100 and 1 μm, respectively.

SEM was used to analyze changes in the microstructure of the sample during the transition from the green body after printing (Figure 7) to the brown body after debinding in an Ar + H_2_ atmosphere (Figure 8).

The boundaries between the printed layers are clearly visible in the green body images. The ceramic filler particles are evenly distributed in the polymer matrix. The presence of small defects, probably related to the shrinkage of the monomer during polymerization or the agglomeration of powder particles, was observed.

After heat treatment, the material undergoes significant changes. As a result of the removal of the organic matrix, the structure becomes visibly more porous, and at low magnification the boundaries of the layers formed at the 3D printing stage remain distinguishable. The structure is less homogeneous and consists of ceramic particles connected through interparticle contacts and possible carbonaceous residue. No large cracks or delamination was observed.

**Figure 9:**
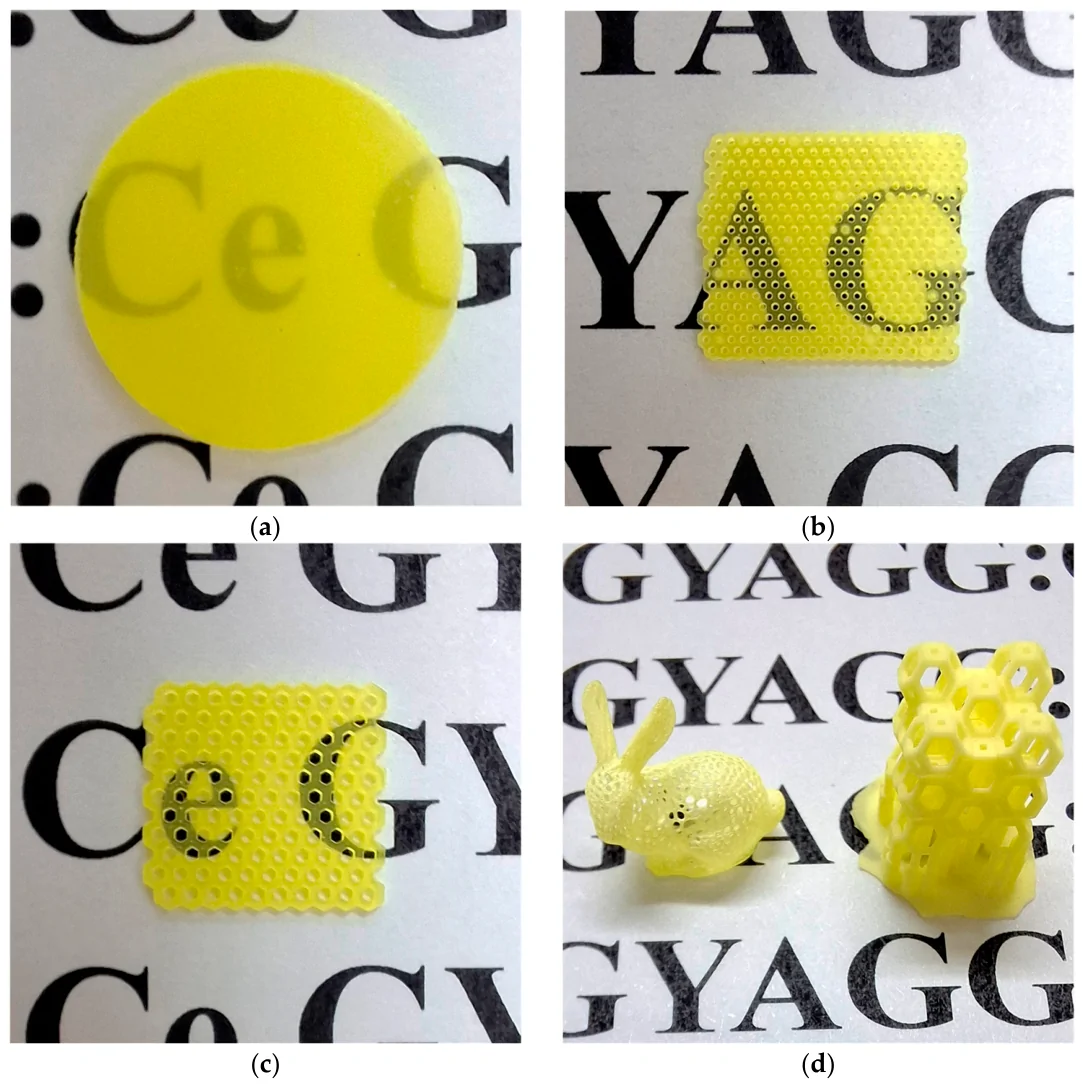
GYAGG:Ce ceramic in the form of a (**a**) 1.2 mm thick disk and (**b**,**c**) honeycomb structures; (**d**) complex-shaped ceramics in the form of a bunny and Kelvin cell.

The green body of complex-shaped GYAGG:Ce/HDDA, as well as a simple disk with a diameter of 20 mm and a thickness of 2 mm, was debound using the 95% Ar + 5% H_2_ schedule described in Section 2. These observations demonstrate the feasibility of the selected schedule for the investigated geometries, but do not establish its universal applicability to arbitrary dimensions or wall thicknesses (Figure 9).

No large cracks, delamination, or macroscopic collapse was observed after debinding or subsequent sintering in the investigated specimens. These observations demonstrate the feasibility of the selected schedule for the investigated geometries, but do not establish its universal applicability to arbitrary dimensions or wall thicknesses.

The full and linear transmittance of the 1.2 mm thick ceramic disk (Figure 9a) at a wavelength of 525 nm was 54.5 and 12%, respectively, demonstrating measurable optical transmission after the thermal treatment (full and in-line transmittance spectra are shown in Figure S7).

For comparison, previously reported GAGG/GYAGG-type transparent ceramics show a broad range of optical transmittance depending on composition, thickness, powder characteristics, and sintering conditions [33,34]. Therefore, the values obtained here demonstrate successful optical transmission but should not be considered a record transparency value.

For the visually translucent honeycomb structures (Figure 9b,c), the mean distances between opposite walls of the hexagonal openings were 257 ± 17 and 448 ± 30 µm, respectively. The structures of the bunny and Kelvin cell (Figure 9d) provide a qualitive demonstration of the successful debinding and sintering of geometrically complex ceramic objects.

## 4. Conclusions

The thermal decomposition behavior of DLP-printed GYAGG:Ce/HDDA green bodies was systematically investigated using non-isothermal TGA/DTG at four heating rates (2, 4, 8 and 20 °C/min), with four independent measurements at each rate. The thermal behavior and kinetics of decomposition green bodies were investigated and showed a complex, conversion-dependent behavior. The principal mass-loss stage occurred at approximately 350–470 °C, while the mean DTG peak shifted from 387.1 ± 10.3 at 2 °C/min to 427.9 ± 15.2 at 20 °C/min. The conversion degree’s dependence on the apparent activation energy was determined by KAS and FWO methods, while the main mass-loss peak was described by the Kissinger model (E_a_ = 200.85 kJ/mol). The MS analysis of the gas phase formed during the thermal decomposition detected signals that were tentatively assigned to water vapor, carbon oxides and fragments of the acrylate organic chains. The KAS and FWO results showed an increase in apparent activation energy with conversion, with the most reliable linear-regression range occurring approximately at α = 0.6–0.8.

This study provided an experimental demonstration of a binder removal schedule based on TGA using 95% Ar + 5% H_2_ for a disk measuring 20 mm × 2 mm and complex-shaped bodies made of GYAGG:Ce. The selected schedule slowed down the heating rate in the main mass-loss zone and was successfully applied to the studied samples without the formation of large cracks, delamination, or macroscopic destruction. Thus, the results show that TGA data can be used for the practical removal of the binder from the studied bodies; however, additional verification is required for parts with significantly different dimensions and wall thicknesses.

## Figures and Tables

**Table 1:** Comparison of instrument-derived and independently measured mass losses.

Heating Rate, °C/min	TG Mass Loss, %	Gravimetric Mass Loss, %	Difference, Percentage Points
2	−21.64 ± 0.47	−22.32 ± 0.13	0.68
4	−22.59 ± 0.48	−22.10 ± 0.29	0.49
8	−21.92 ± 0.39	−23.62 ± 0.61	1.70
20	−21.68 ± 0.30	−22.92 ± 1.20	1.24

**Table 2:** Mean DTG peak temperatures at different heating rates.

Heating Rate, °C/min	Main Mass-Loss Peak, °C
2	387.1 ± 10.3
4	395.6 ± 9.6
8	408.9 ± 6.5
20	427.9 ± 15.2

## Data Availability

The original contributions presented in this study are included in the article/Supplementary Material. Further inquiries can be directed to the corresponding authors.

## Supplementary Materials

The following supporting information can be downloaded at https://www.mdpi.com/article/10.3390/polym18202554/s1. Figure S1: FTIR OMNIC library-search results for pure HDDA; Figure S2: (a) Particle size distribution; (b) SEM image of the initial GYAGG:Ce powder; Figure S3: XRD spectra of the initial GYAGG:Ce powder; Figure S4: TGA and DTG curves of the GYAGG:Ce/HDDA green bodies recorded under (a) air; (b) argon; (c) Ar + H_2_ (50%); Figure S5: FTIR spectra of pure HDDA and resinous condensates collected after pyrolysis under Ar + H_2_, Ar, and vacuum; Figure S6: FTIR OMNIC library-search results for resinous condensates collected after pyrolysis under (a) Ar + H_2_; (b) Ar; (c) vacuum; Figure S7: (a) Full and (b) linear transmittance spectra for 1.2 mm thick bulk ceramic disk.

## Abbreviations

The following abbreviations are used in this manuscript:

DLP	Digital light processing
DSC	Differential scanning calorimetry
DTG	Derivative thermogravimetry
FWO	Flynn–Wall–Ozawa
HDDA	1,6-hexanediol diacrylate
KAS	Kissinger–Akahira–Sunose
MS	Mass spectrometry
SEM	Scanning electron microscopy
TGA	Thermogravimetric analysis
TPO	Diphenyl(2,4,6-trimethylbenzoyl)phosphine oxide
