# Supplementary material for: Thermal Analysis of 1,6-Hexanediol Diacrylate-Based Green Bodies Containing GYAGG:Ce Fabricated by DLP 3D Printing
Source: Polymers (Basel). 2026 Oct 16;18(20):2554. doi: 10.3390/polym18202554 (PMC13709203; doi:10.3390/polym18202554)
Supplement: Supplementary file 1 [file polymers-18-02554-s001.zip › polymers-4525185-supplementary.pdf]

Supplementary Materials for

Thermal analysis of 1,6-hexanediol diacrylate-based green bodies containing GYAGG:Ce fabricated by DLP 3D printing

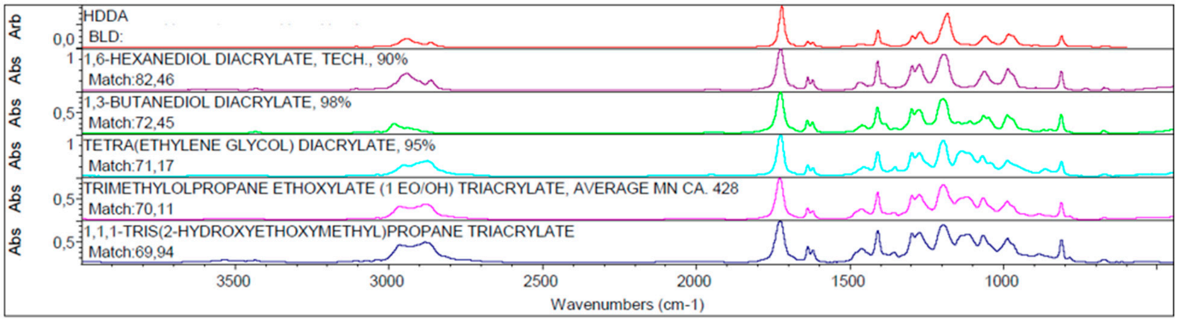

Figure S1. FTIR OMNIC library-search results for pure HDDA.

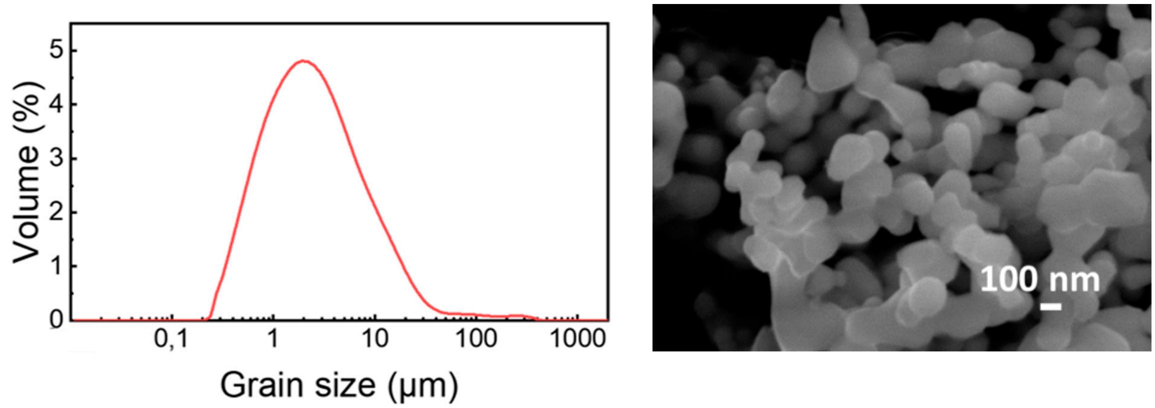

(**a**) (**b**)

Figure S2. (a) Particle size distribution; (b) SEM image of the initial GYAGG:Ce powder.

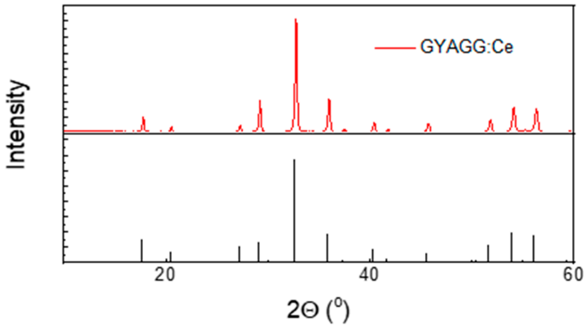

Figure S3. XRD spectra of the initial GYAGG:Ce powder.

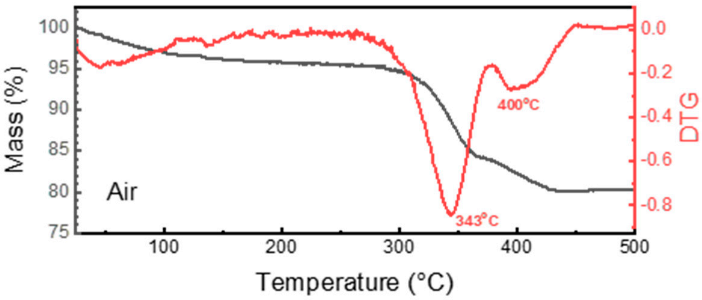

(**a**)

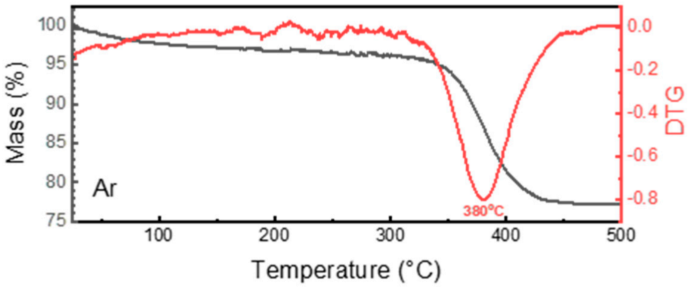

(**b**)

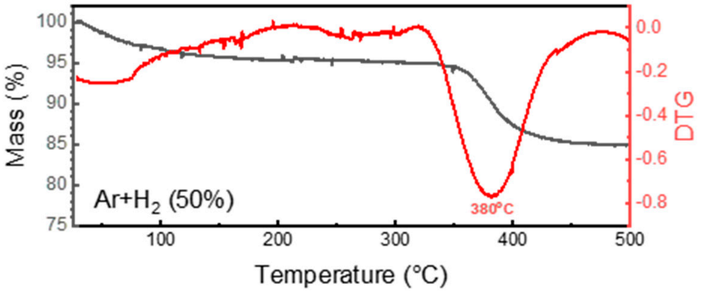

(**c**)

Figure S4. TGA and DTG curves of the GYAGG:Ce/HDDA green bodies recorded under (a) air; (b) argon; (c) Ar+H_2 (50%).

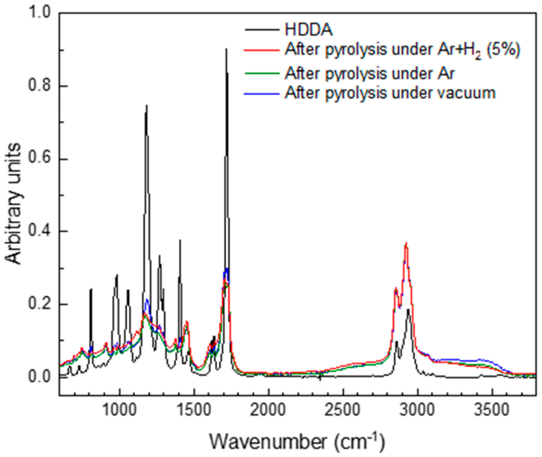

Figure S5. FTIR spectra of pure HDDA (black line) and resinous condensates collected after pyrolysis under Ar+H_2, Ar, and vacuum by red, green and blue lines respectively.

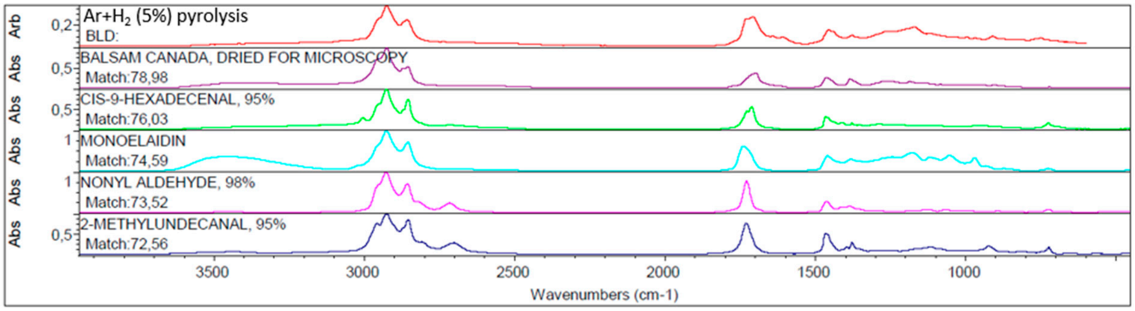

(**a**)

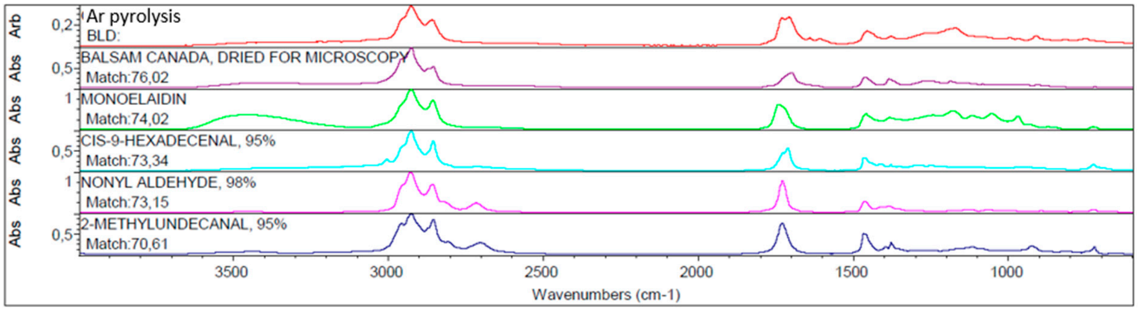

(**b**)

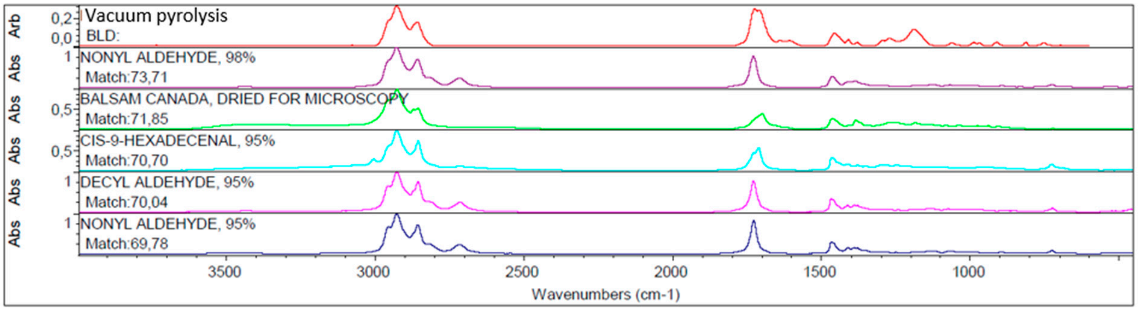

(**c**)

Figure S6. FTIR OMNIC library-search results for resinous condensates collected after pyrolysis under (a) Ar+H_2; (b) Ar; (c) vacuum.

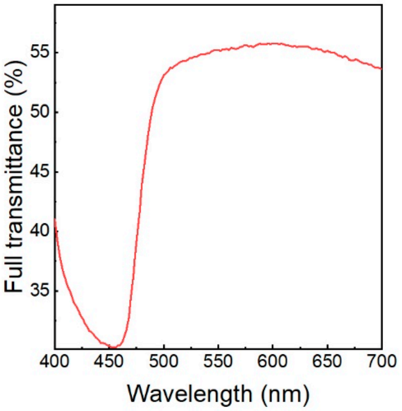

(**a**)

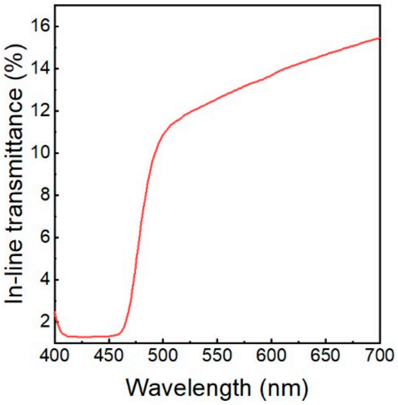

(**b**)

Figure S7. (a) Full and (b) linear transmittance spectra for 1.2 mm thick bulk ceramic disk.
